# Mechanisms Governing Photon-Pair Generation and Emission Directionality in Quantum Metasurfaces

**DOI:** 10.1021/acsphotonics.6c01607

**Published:** 2026-10-13

**Authors:** Alberto Paniate, Ivano Ruo-Berchera, Francesco Monticone

**Affiliations:** † Quantum Metrology and Nanotechnologies Division, 83364 INRiM, Strada delle Cacce 91, 10135 Torino, Italy; ‡ Friedrich-Alexander-Universität Erlangen-Nürnberg, Staudtstr. 7, 91058 Erlangen, Germany; § 5922 Max Planck Institute for the Science of Light, Staudtstr. 2, 91058 Erlangen, Germany; ∥ School of Electrical and Computer Engineering, Cornell University, Ithaca, New York 14853, United States

**Keywords:** spontaneous parametric down-conversion, nonlinear metasurfaces, quantum metasurfaces, quasi-normal modes, nano-optics, photon-pair generation, emission directionality, efficiency

## Abstract

Metasurfaces are emerging as a promising platform for photon-pair generation through spontaneous parametric down-conversion, thanks to their compactness, integrability, and intrinsic multifunctionality, which enables the engineering of complex quantum states. However, their full potential remains only partially exploited because the physical mechanisms governing the key properties of the generated photon pairs, such as generation efficiency and emission directionality, are not yet fully understood. As a result, metasurface designs and experimental configurations are often optimized through trial and error procedures. Here, we theoretically investigate the main mechanisms that control photon-pair generation and detection by studying how different pump configurations and measurement geometries affect the generation efficiency, emission directionality, and collection efficiency of emitted photon pairs. This framework allows us to interpret existing experimental results and provide general guidelines for the design of metasurfaces and the choice of experimental configurations in future experiments. Finally, we show that substrate thickness and multilayer configurations represent additional degrees of freedom for quantum metasurface design and can be engineered to enhance the generation efficiency and control the emission directionality, providing a new route for the optimization of photon-pair sources based on metasurfaces.

## Graphical abstract

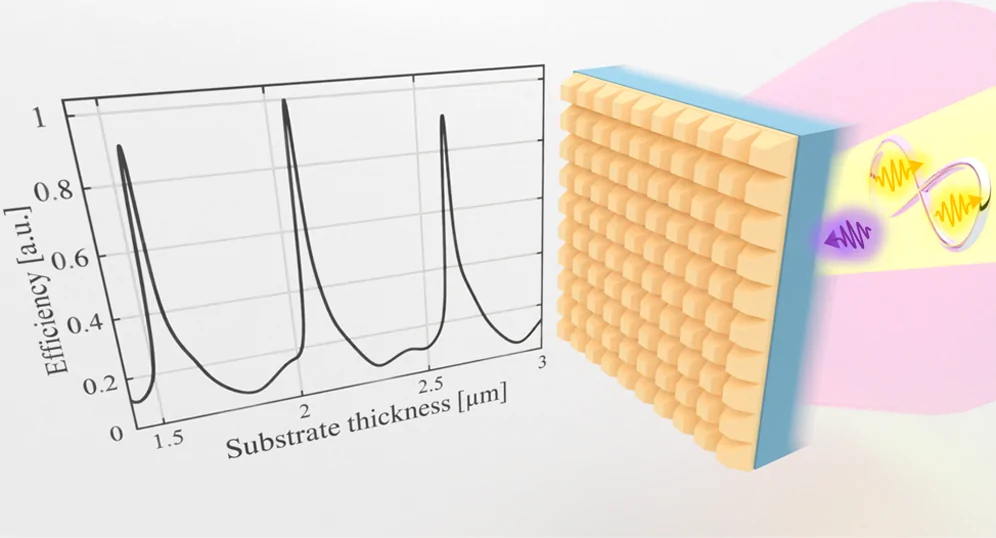

## Introduction

Traditionally, spontaneous parametric down-conversion (SPDC) has been performed in bulk noncentrosymmetric crystals, where the millimeter-scale interaction length between the pump field and the nonlinear medium enables efficient generation of entangled photon pairs, commonly referred to as signal and idler photons. However, this long interaction length also imposes stringent phase-matching constraints, thereby limiting the accessible properties of the generated photon pairs and restricting the choice of suitable dispersive materials. In recent years, micrometer- and nanometer-thick nonlinear films have attracted considerable interest as compact and integrable SPDC sources with relaxed longitudinal phase-matching conditions.^1–6^ Nevertheless, the intrinsically low conversion efficiency of ultrathin nonlinear media has motivated the development of structured nonlinear systems, in which the resonances supported provide strong field enhancement and thereby significantly increase the photon-pair generation efficiency. SPDC has been demonstrated both in single nanoresonators^7–10^ and in metasurfaces, i.e., periodic arrays of nanoresonators.^11–21^

Beyond efficiency enhancement, nonlinear metasurfaces offer a large number of degrees of freedom. This flexibility enables multifunctional quantum-light sources with properties that are difficult to achieve in bulk crystals, such as engineered spatial emission,^12,15,17^ tunable and arbitrary polarization Bell-state generation,^14,20,21^ and cluster-state generation.^13^ Although their efficiencies remain generally lower than those of conventional bulk sources, the multifunctional properties achievable, together with compactness and integrability, represent their main advantages for quantum-light generation.

However, the full exploitation of this multifunctionality requires accurate design and optimization strategies tailored to the specific target properties. At present, this progress is strongly limited by the lack of a comprehensive theoretical framework describing the mechanisms that govern quantum emission from nonlinear metasurfaces. As a result, key photon-pair properties, such as generation efficiency and emission directionality, as well as the optimal collection geometry, are often difficult to predict and are still largely determined through trial and error approaches.

Here, we take an important step forward in this direction by theoretically investigating, within a quasi-normal-mode (QNM) framework,^10,22,23^ how different pump directions and detection geometries affect the photon-pair generation efficiency and the collection of the emitted photons. The theoretical predictions are compared with existing experimental results obtained from a lithium-niobate (LN) metasurface fabricated on a glass substrate,^11^ providing a physical interpretation of the observed behavior.

Motivated by the crucial role of substrate-induced effects emerging from this analysis, we investigate both the generation efficiency and the emission directionality first within a semi-infinite substrate approximation, which isolates the intrinsic response of the metasurface by neglecting finite-thickness effects, and then for substrates with finite thickness and multiple layers. By addressing these effects, which remain largely unexplored in SPDC metasurfaces, our analysis opens new routes to enhance photon-pair generation and tailor the emission properties of quantum metasurfaces, similar to recent substrate-interference strategies used to enhance second-harmonic generation in two-dimensional materials.^24,25^

## Results and Discussion

### Theoretical Framework and Mode Structure

To illustrate the proposed theoretical framework we consider a realistic example. The investigated metasurface consists of an infinite-periodic array, with periodicity of 900 nm, of truncated pyramids (the complete geometrical and nonlinear parameters are provided in Section 1 of the Supporting Information) and is excited by a pump field at wavelength λ_*p*_ = 788 nm.^11^ The metasurface supports several resonances, shown in Figure 1 and labeled with numbers 1–3. Owing to their intrinsically leaky nature, these resonances are commonly referred to as QNMs.^26,27^ The red dots in Figure 1 indicate the different QNMs together with their corresponding quality factors, defined as *Q* = ω/(2 ζ), where ω and ζ are respectively the real and imaginary parts of the complex eigenfrequency ω̃ = ω – *iζ*. Here, ω determines the resonance frequency, while ζ describes the radiative leakage rate of the mode.

**Figure 1:**
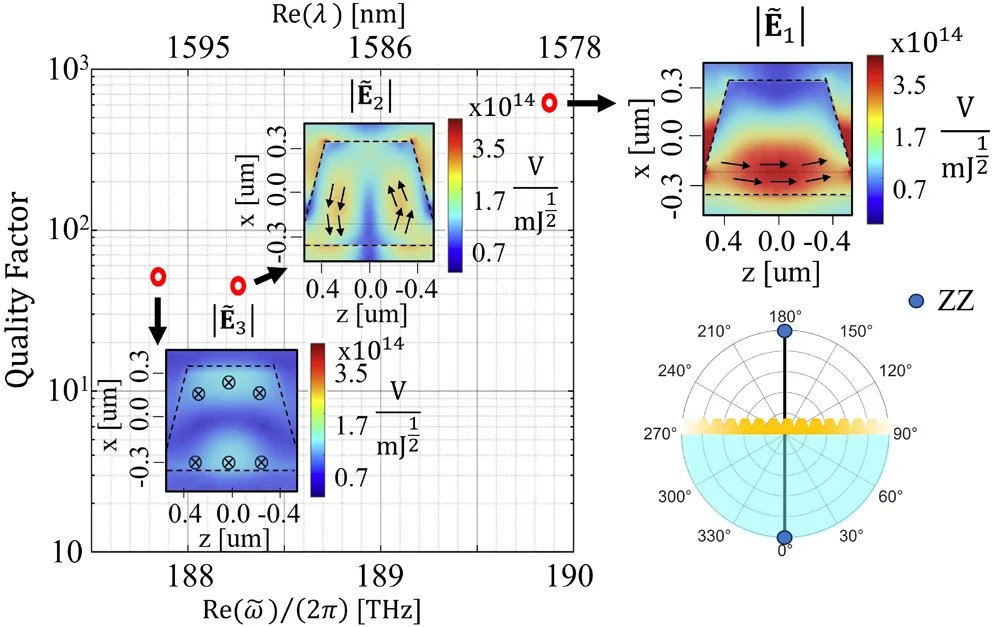
Quasi-normal modes (QNMs) supported by the metasurface. The metasurface, deposited on a glass substrate, supports three QNMs, characterized by complex eigenfrequencies ω̃ = ω – *iζ*, where ω is the resonance frequency and ζ is the leakage rate. The QNMs are indicated by red dots, together with their resonance frequencies and quality factors, defined as *Q* = ω/(2ζ). The insets show the corresponding electric near-field distributions inside a single unit-cell of the infinite metasurface, with black arrows and crossed circles indicating the local electric-field polarization. The right panel highlights the dominant mode, labeled QNM_1_, which exhibits the strongest electric field inside the nonlinear material. Its near-field distribution is shown in the upper panel, while the corresponding far-field emission pattern is shown in the lower panel as a function of the polar angle.

The QNMs are shown together with their electric near-field distributions inside a single unit-cell of the metasurface |**Ẽ**|. Each QNM is normalized according to QNMs theory^28^ and detailed in Supporting Information Sections 1–2. The black arrows and crossed circles indicate the local polarization of the electric field. Among the different modes, QNM_1_, whose resonance wavelength is closest to the degenerate SPDC wavelength, (2λ_*p*_ = 1576 nm), exhibits the largest near-field amplitude inside the nonlinear material. Moreover, its electric field is predominantly oriented along the optical axis of LN, corresponding to the *z*-direction, for which the nonlinear tensor component χ_*zzz*_^(2)^ is the largest.

For these reasons, in the following, we use QNM_1_ as a representative resonant channel to construct a reduced description of the SPDC response and isolate the main mechanisms governing the substrate-dependent generation and directionality of photon pairs.

The QNMs shown in Figure 1 are retrieved with zero Floquet wave-vector, i.e., zero parallel component of the wave-vector of the field, which describes the normal-emission channel. The main analysis is restricted to this channel. The validity and limitations of this approximation are examined in Supporting Information Section 3.

Below the near-field profile of QNM_1_ in Figure 1, the angular dependence of the corresponding complex electric far-field, |**Ẽ**(θ)|, is shown. For the geometry considered here, QNM_1_ exhibits approximately equal normal far-field electric-field amplitudes in the air and substrate half-spaces. We stress that equal electric-field amplitudes do not imply equal emitted photon fluxes, owing to the different refractive indices of the two surrounding media. The far-field is predominantly *z*-polarized, consistently with the dominant polarization of the QNM near field inside the LN resonators. Therefore, in the following analysis, we restrict the discussion to the *z*-polarized contribution of QNM_1_.

The contribution of the QNM pair QNM_1_–QNM_1_ to the SPDC photon-pair detection rate is given by^10,22,23^

1 $$|{\mathrm{T̃}}_{\mathrm{is}}{|}^{2}={\left|\xi \left(\omega_{s}\right){Ẽ}_{z}\left(\theta_{i}\right){Ẽ}_{z}\left(\theta_{s}\right)\right|}^{2}$$

where *T̃*_is_ is the complex two-photon amplitude, namely the probability amplitude associated with the joint detection of the idler and signal photons. The signal and idler angular frequencies are ω_s_ and ω_i_ = ω_p_ – ω_s_, respectively, where ω_p_ is the pump angular frequency. The two photons are detected in the far-field along the directions of polar angles θ_i_ and θ_s_, respectively, with polarization along the *z*-axis. In the normal-emission case considered here, the relevant far-field amplitudes are evaluated along the normal-emission channels: θ = 0° on the substrate side and θ = 180° on the air side, as shown in Figure 1.

While the QNMs are intrinsic properties of the metasurface and therefore independent of the excitation field, the contribution of a QNM pair is weighted by the nonlinear modal overlap coefficient ξ­(ω_s_)­

2 $$\xi \left(\omega_{s}\right)=\frac{\sum_{\alpha ,\beta ,\gamma }\int dr_{0}\chi_{\alpha \beta \gamma }^{\left(2\right)}\left(r_{0}\right){Ẽ}_{\alpha }\left(r_{0}\right){Ẽ}_{\beta }\left(r_{0}\right)E_{p,\gamma }\left(r_{0}\right)}{\left(\omega_{p}-\omega_{s}-\mathrm{\omega ̃}\right)\mathrm{\omega ̃}\left(\omega_{s}-\mathrm{\omega ̃}\right)\mathrm{\omega ̃}}$$

where the numerator quantifies the nonlinear interaction inside the LN resonators through the spatial overlap between the nonlinear susceptibility tensor χ^(2)^, the QNM near-field **Ẽ** along directions α and β, and the pump field **E**_p_ along direction γ. The detuning term in the denominator, instead, accounts for the spectral mismatch between the generated photons (ω_s_, ω_i_) and the complex eigenfrequency ω̃ of QNM_1_. For the complete model see Supporting Information Section 1.

Within this theoretical framework, we analyze how different excitation and collection geometries affect the detected photon-pair rate.

### SPDC Efficiency and Directionality with Different Excitation-Collection Geometries and Semi-Infinite Substrate

We consider a plane-wave excitation incident on the metasurface supported by a semi-infinite glass substrate. The theoretically calculated number of detected photon pairs is integrated over the spectral range from 1550 nm to 1600 nm, and the resulting count rates are then compared for the four different excitation and detection geometries illustrated in Figure 2.

**Figure 2:**
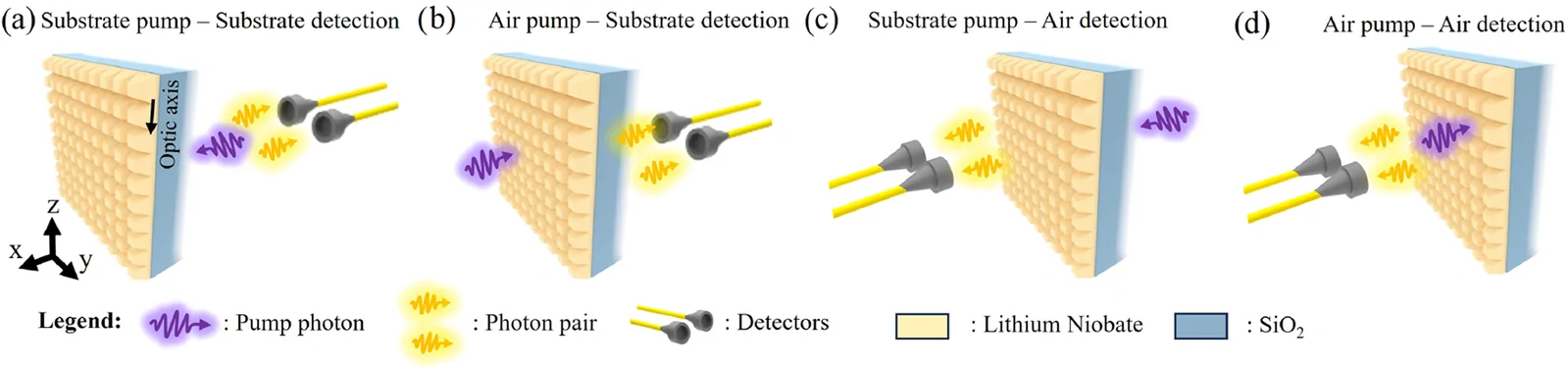
Schematic representation of the four pump and detection geometries considered in this work. A lithium-niobate metasurface with the optic axis aligned along the *z*-axis is deposited on a glass substrate^11^ and is pumped by a laser under different excitation and collection configurations. (a) Pump incident from the substrate side and photon-pair detection from the substrate side. (b) Pump incident from the air side and detection from the substrate side. (c) Pump incident from the substrate side and detection from the air side. (d) Pump incident from the air side and detection from the air side.

The metasurface is excited either from the substrate side or from the air side and, for each excitation configuration, photon-pair collection is analyzed both from the air side (θ_s_ = θ_i_ = 180°) and from the substrate side (θ_s_ = θ_i_ = 0°). Experimentally, the highest enhancement, corresponding to approximately a factor of 20 compared to a thin film of equal thickness, was observed for the reflection configuration (a). By contrast, configurations (b) and (c), referred to as transmission geometries, since the photon pairs are collected along the pump propagation direction, showed no measurable enhancement. Although configuration (d) was not experimentally investigated, our analysis predicts that low enhancement is expected in this case as well, as discussed later.

Importantly, changing the pumping or detection geometry does not modify the intrinsic QNM properties or their eigenfrequencies. Therefore, the difference between the investigated configurations arises from two configuration-dependent factors: the pump-dependent nonlinear overlap |ξ|^2^, particularly through the numerator of eq 2, and the portion of the QNM far-field that is selected by the detection geometry, which enters through the factor |*Ẽ*_*z*_(θ_s_)*Ẽ*_*z*_(θ_i_)|^2^.

Figure 3 compares configurations (a) and (b) in which the photon pairs are collected from the same side, while the pump is incident either from the substrate side or from the air side. Since the collection side is unchanged, the QNM far-field contribution in eq 1 remains the same, while the numerator in eq 2 changes through the different pump-field distributions inside the LN resonators. The corresponding pump electric-field amplitudes inside one unit-cell are shown in Figure 3(a,b), revealing clear differences between the two excitation configurations. These differences arise from the asymmetry of the resonators and the different pump fields experienced by the metasurface. In fact, for substrate-side excitation, the metasurface is predominantly driven by the field transmitted through the glass substrate. In contrast, for air-side excitation, the pump field inside the LN resonators results from the interference between the incident field and the field reflected and scattered by the metasurface–substrate system. As a consequence, the two pumping configurations generate distinct spatial pump-field distributions inside the nonlinear material.

**Figure 3:**
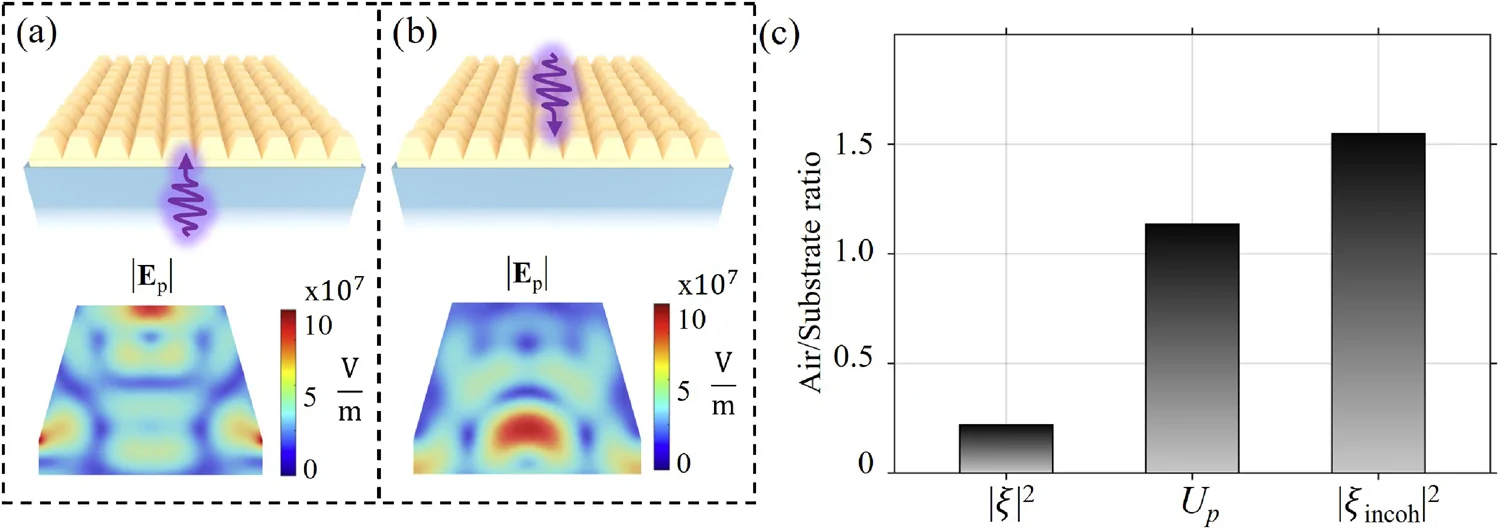
Effect of the pump direction on the nonlinear modal overlap. (a, b) Pump electric-field amplitude |**E**_p_| inside one unit-cell of the LN metasurface for substrate-side pumping (a) and air-side pumping (b). (c) Air-side/substrate-side ratios of the coherent nonlinear-overlap contribution |ξ|^2^, the integrated pump-field intensity inside the nonlinear material *U*_p_, and the incoherent overlap |ξ_incoh_|^2^. All values are evaluated for equal incident pump photon flux in the two excitation configurations.

The effect of the pump direction is summarized in Figure 3(c), which reports the air-side/substrate-side ratios of three relevant quantities. The first bar shows the ratio of the modal overlap contribution |ξ|^2^. Air-side pumping yields a value of only ∼0.2 relative to substrate-side pumping, indicating that substrate-side excitation enhances the SPDC generation efficiency by approximately a factor of 5, in qualitative agreement with the experimental observations.

To determine whether this enhancement simply originates from a larger pump intensity inside the nonlinear material, rather than an enhanced modal overlap, the second bar shows the ratio of the integrated pump-field intensity inside the LN resonators, defined as

$$U_{p}=\int {|E}_{p}\left(r_{0}\right){|}^{2}dr_{0}$$ 3

The ratio of *U*_p_ is close to unity, indicating that comparable amounts of pump-field intensity are coupled into the nonlinear material for the two excitation directions.

The third bar reports the ratio of the incoherent overlap, |ξ_incoh_|^2^, obtained by taking the absolute value of the integrand in eq 2 before performing the spatial integration. This quantity is larger for air-side pumping, indicating that the intensity overlap between the pump field and the nonlinear source distribution associated with QNM_1_ is not reduced in this configuration. This result is also qualitatively consistent with the field distributions shown in Figure 3(a,b) and Figure 1. In fact, for air-side excitation, the pump-field amplitude exhibits a stronger concentration in the lower central region of the LN resonator, where QNM_1_ also displays a pronounced near-field amplitude.

Therefore, the strong suppression of |ξ|^2^ for air-side excitation cannot be attributed either to a lower pump intensity inside the LN resonators or to a weaker intensity overlap. Instead, it originates from stronger phase cancellations within the coherent nonlinear-overlap integral. This demonstrates that the pump direction controls the phase and symmetry matching between the pump field and the QNM nonlinear source distribution, thereby determining the photon-pair generation efficiency.

We next compare configurations (a) and (c), where the pump is incident from the substrate side in both cases, while the photon pairs are collected either from the substrate side (θ_s_ = θ_i_ = 0°) or from the air side (θ_s_ = θ_i_ = 180°). Since the excitation geometry is unchanged, the pump-field distribution inside the LN resonators is the same for the two configurations. Consequently, the overlap factor |ξ|^2^ is also unchanged, and the difference in the detected photon-pair rate originates from the far-field emission and collection factors in eq 1.

As shown in Figure 1, the far-field electric-field amplitude of QNM_1_, calculated for the metasurface on a semi-infinite glass substrate, is symmetric between the air and substrate half-spaces. However, this polar plot represents the QNM electric-field amplitude, not directly the emitted photon flux. In fact, because of the different refractive indices of the two media, comparable far-field amplitudes do not correspond to equal photon fluxes. The final air-side to substrate-side photon-pair emission ratio is approximately 0.44 (see Supporting Information Section 1). Consequently, configuration (a) exhibits a higher detected photon-pair rate than configuration (c), in qualitative agreement with the experimental observations.^11^

We note, however, that this comparison is based on an infinite-periodic-metasurface approximation. Although Floquet-periodic boundary conditions are commonly used to describe metasurfaces, experiments probe only a finite region of the structure because of the finite pump-beam waist. Finite aperture effects are known to modify resonant properties such as the quality factor, radiative outcoupling, far-field emission pattern, and edge-related leakage.^29,30^

In the original experiment,^11^ the pump beam had a finite width of approximately 6 μm, corresponding to the excitation of only 7–8 unit-cells. To assess the impact of this finite-excitation aperture, we simulated a finite array of nanoresonators with a comparable number of coherently excited unit-cells (Supporting Information Section 4). In this case, the far-field emission becomes strongly asymmetric and is predominantly directed toward the substrate, as a result of the combined effect of the finite illuminated area and the refractive-index mismatch between the air and substrate half-spaces, consistent with the known asymmetric emission of finite antennas and quantum emitters above substrates.^31,32^ The resulting air-side to substrate-side photon-pair emission ratio is reduced below 0.1, in agreement with the experimental observation of no measurable emission in this configuration.

We emphasize that this finite-array analysis is specific to the experimental conditions considered here, where only a few unit-cells are effectively excited by the focused pump beam. For larger illumination areas, the infinite-periodic approximation is expected to become increasingly accurate. Nevertheless, finite-excitation aperture effects can substantially impact the emission directionality and collection efficiency and should therefore be carefully considered when drawing quantitative conclusions.

Finally, although configuration (d) was not investigated experimentally, our model predicts it to yield the lowest efficiency. This is because it combines a reduced nonlinear overlap, |ξ|^2^, with inefficient collection due to the weak emission of the mode in the corresponding detection direction.

### SPDC Efficiency and Directionality with a Finite Substrate

While the previous analysis was performed by considering a semi-infinite substrate, thereby neglecting etalon effects induced by multiple reflections in the substrate, we now consider a finite-thickness substrate, as shown in Figure 4(a), and study its effect on the generation efficiency and emission directionality. In the following, we focus on the case of a plane-wave pump incident from the substrate side given the higher efficiency found in the previous analysis.

**Figure 4:**
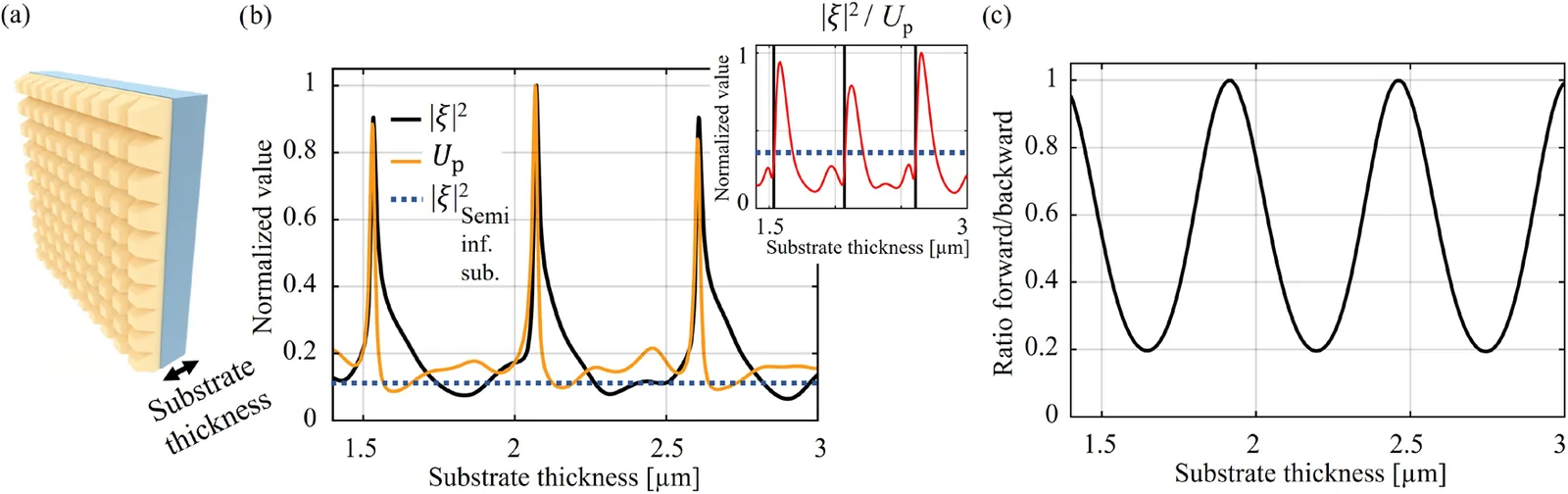
Finite-substrate effects on SPDC generation and emission directionality. (a) Schematic of the finite-substrate geometry, where the substrate thickness is varied. (b) Normalized nonlinear modal overlap contribution, |ξ|^2^, and integrated pump-field intensity inside the nonlinear material, *U*_p_, as a function of the substrate thickness. The horizontal blue dashed line indicates the value of |ξ|^2^ obtained for a semi-infinite substrate. The inset shows the normalized ratio |ξ|^2^/*U*_p_; the black vertical lines mark the substrate thicknesses corresponding to the main |ξ|^2^ peaks, while the blue dashed line indicates the semi-infinite-substrate value of the same ratio. All curves are independently normalized to their maximum value. For each layer configuration, the QNMs and their normalizations were calculated independently. (c) Ratio between air and substrate photon-pair emission as a function of the substrate thickness.

Figure 4(b) shows the nonlinear-overlap contribution |ξ|^2^ as a function of the substrate thickness. The response exhibits a sequence of pronounced and narrow maxima, with variations approaching 1 order of magnitude. In particular, the main efficiency peaks closely follow the behavior of the integrated pump-field intensity inside the nonlinear material, *U*_p_, shown by the orange curve.

This modulation originates from finite-substrate interference effects. Since the metasurface period is 900 nm, larger than the pump wavelength inside the substrate, the pump field scattered by the metasurface can be reflected into several diffraction orders in the glass. These diffraction orders undergo multiple reflections at the substrate interfaces and interfere constructively or destructively depending on the substrate thickness. As a result, the pump field coupled into the LN resonators becomes strongly thickness-dependent, leading to the observed modulation of |ξ|^2^. A complete analytical description of the periodicity and its relation to the relevant diffraction orders is provided in section 5 of the Supporting Information.

The constructive recoupling of reflected or weakly coupled diffraction orders through multiple interference inside the substrate enables an enhancement by almost 1 order of magnitude compared to the semi-infinite substrate case, whose efficiency is indicated by the horizontal dashed blue line in Figure 4(b).

Although this enhanced coupling is beneficial for reducing unwanted pump reflection and increasing the nonlinear interaction, the largest efficiency peaks are mainly associated with a larger integrated pump-field intensity inside the LN resonators. This aspect is important for practical operation, since large field intensities confined in the nonlinear material may increase the risk of optical damage, heating, or other intensity-induced degradation mechanisms. It is therefore useful to evaluate not only the absolute overlap contribution |ξ|^2^ but also the generation efficiency normalized to the pump-field intensity stored in the nonlinear material. To this aim, the inset in Figure 4(b) shows the ratio |ξ|^2^/*U*_p_. This quantity isolates the part of the efficiency enhancement that does not simply arise from an increased pump intensity inside the nonlinear material. Indeed, the substrate thickness modifies not only the total amount of pump field coupled into the metasurface but also its spatial distribution and phase inside the LN resonators, modifying the final generation efficiency.

This mechanism is reflected in the oscillatory behavior of |ξ|^2^/*U*_p_, which exhibits maxima and minima differing by almost 1 order of magnitude. Moreover, the maxima of this normalized efficiency are slightly shifted with respect to the maxima of *U*_p_, shown with the black vertical lines. In particular, there is a maximum enhancement of approximately a factor of 3 compared to the semi-infinite glass substrate case, indicated with blue line. This demonstrates that substrate-thickness engineering can improve the photon-pair generation efficiency by optimizing the coherent nonlinear overlap, rather than simply increasing the pump intensity inside the metasurface.

These results suggest a useful optimization strategy for damage-limited operation: instead of maximizing only |ξ|^2^, one can search for substrate thicknesses that maximize the photon-pair generation efficiency per pump-field intensity stored in the nonlinear material. In this way, the SPDC efficiency can be enhanced through improved coherent overlap, without necessarily increasing the local pump-field intensity inside the metasurface.

Finally, Figure 4(c) shows the ratio between photon-pair emission in the air and substrate directions as a function of the substrate thickness. This ratio exhibits pronounced smooth oscillations, originating from Fabry–Pérot-like interference effects inside the finite substrate. Indeed, at the signal and idler wavelengths, the metasurface period is smaller than the wavelength in both surrounding media, so that only the zeroth diffraction order is propagating and all higher diffraction orders are evanescent. Furthermore, the modulation is enhanced by the high-*Q* resonance of the metasurface, which generates spectrally narrow photon pairs with a correspondingly long coherence length. As a result, the different reflected contributions inside the substrate can interfere coherently and modify the relative emission into the two directions.

Although the present analysis is limited to a finite glass substrate of a few micrometers surrounded by air, it suggests a more general strategy based on realistic multilayer substrates. To support this perspective, we investigate an elementary multilayer configuration in Section 6 of the Supporting Information, showing that substrate engineering can provide an experimentally feasible route to enhance and tailor SPDC from nonlinear metasurfaces.

## Conclusions

In this work, we have developed a theoretical framework that identifies the main mechanisms that govern photon-pair generation and collection in a nonlinear metasurface and used it to provide a physical interpretation of existing experimental results. Our analysis extends the use of a QNM-based description of SPDC, previously developed for single nanoresonators,^10,23^ to a periodic metasurface supporting high-*Q* resonances. Within this framework, the different excitation and collection geometries are understood through two main contributions: the pump-dependent coherent nonlinear modal overlap and the direction-dependent far-field emission of the selected resonant QNMs. Furthermore, the present analysis should be regarded as a first step toward a more complete multimode description of SPDC in periodic metasurfaces, in which the contributions of multiple QNMs, together with their angular dispersion and dependence on the Floquet wave-vector, are treated consistently in order to reconstruct the full angle-resolved biphoton state.

Our results also highlight the important role of substrate-related effects in the SPDC metasurfaces. While finite-substrate effects have so far received little attention in this context, we show that substrate thickness can act as an additional design parameter for controlling both the generation efficiency and emission directionality. In particular, finite-substrate interference and diffraction-order recoupling can enhance the pump coupling to the metasurface and modify the coherent nonlinear overlap. Importantly, the efficiency can be improved not only by increasing the pump-field intensity inside the nonlinear material but also by optimizing the nonlinear overlap for a comparable stored pump intensity. This provides a promising route toward enhanced operation under practical constraints associated with optical damage, heating, and other intensity-induced degradation mechanisms. The proof-of-principle multilayer example presented in the Supporting Information Section 6 further indicates that the same physical mechanism can be translated into more realistic substrate geometries, provided that the metasurface period, pump wavelength, and refractive-index environment are designed together.

Although the quantitative enhancement factors reported here are specific to the investigated LN metasurface, the physical mechanisms identified by our analysis suggest general guidelines for resonant SPDC metasurfaces. First, the excitation configuration should be optimized by maximizing the coherent nonlinear overlap. Second, the collection geometry should be selected by considering the QNM far-field emission and the photon flux into the relevant surrounding media, while finite-excitation-aperture effects should be accounted for whenever only a limited number of unit-cells are effectively illuminated. Third, when substrate-induced interference is exploited, the substrate thickness and refractive-index environment should be codesigned with the metasurface period and pump wavelength so that the relevant pump diffraction channels are efficiently recoupled to the nonlinear resonators. Finally, for damage-limited operation, a useful optimization target is the generation efficiency per pump-field intensity stored in the nonlinear material, as quantified here by |ξ|^2^/*U*_p_.

These design principles provide a foundation for future optimization and inverse-design strategies^33^ aimed at realizing more efficient, directional, and experimentally robust quantum metasurface sources.

## Supplementary Material





## Notes

Geometrical and nonlinear parameters of the metasurface (S1.1); theoretical model and QNMs normalization (S1.2); transmittance retrieved with QNMs (S2); QNMs with nonzero Floquet boundary conditions (S3); finite size metasurface simulation (S4); substrate effects induced by diffraction channels reflection (S5); proof of principle of a multilayer design (S6) (PDF)
Images and references of Supplementary Material (ZIP)
