# Supplementary material for: Mechanisms Governing Photon-Pair Generation and Emission Directionality in Quantum Metasurfaces
Source: ACS Photonics. 2026 Oct 13;13(21):6442–8. doi: 10.1021/acsphotonics.6c01607 (PMC13741989; doi:10.1021/acsphotonics.6c01607)
Supplement: Supplementary file 1 [file ph6c01607_si_001.pdf]

Supporting Information

Mechanisms governing photon-pair generation and emission directionality in quantum metasurfaces

Alberto Paniate,[1,2,3,*] Ivano Ruo-Berchera,[1,†] and Francesco Monticone[4,†]

[1] Quantum metrology and nano technologies division, INRiM, Strada delle Cacce 91, 10135 Torino, Italy
[2] Friedrich-Alexander-Universität Erlangen-Nürnberg, Staudtstr. 7, 91058 Erlangen, Germany
[3] Max Planck Institute for the Science of Light, Staudtstr. 2, 91058 Erlangen, Germany
[4] School of Electrical and Computer Engineering, Cornell University, Ithaca, New York 14853, USA

[*] Corresponding author: alberto.paniate@mpl.mpg.de [†] Equal last author contribution.

1 Materials and Computational Methods

1.1 Geometrical and nonlinear parameters of the metasurface

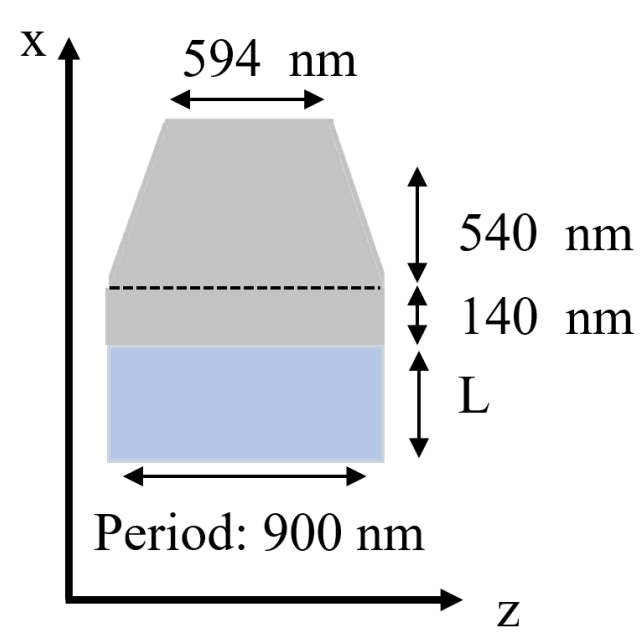

Figure S1: *Geometrical parameters of a single unit-cell of the lithium niobate (LN) metasurface*

In Fig. S1 the unit cell is depicted. It consists of a truncated pyramid with a base length equal to the metasurface period (900 nm), a top base length of 594 nm, and a height of 540 nm, called metasurface B in the original paper.[1] The truncated pyramids rest on a residual layer with an estimated height of 140 nm, which is supported by a glass substrate of height L. The nanostructures are made of lithium niobate (LN), a material well-known for its high second-order nonlinear susceptibility $\chi^{(2)}$. The polarization response, in the laboratory reference frame, can be described as:[1]

$$
\begin{aligned}
P_x^{\mathrm{NL}}(\omega_p) &= 4\varepsilon_0\Big[d_{31}\big(E_x(\omega_s)E_z(\omega_i)+E_z(\omega_s)E_x(\omega_i)\big)-d_{22}\big(E_x(\omega_s)E_y(\omega_i)+E_y(\omega_s)E_x(\omega_i)\big)\Big],\\
P_y^{\mathrm{NL}}(\omega_p) &= 4\varepsilon_0\Big[d_{22}\big(E_y(\omega_s)E_y(\omega_i)-E_x(\omega_s)E_x(\omega_i)\big)+d_{31}\big(E_y(\omega_s)E_z(\omega_i)+E_z(\omega_s)E_y(\omega_i)\big)\Big],\\
P_z^{\mathrm{NL}}(\omega_p) &= 4\varepsilon_0\Big[d_{31}\big(E_x(\omega_s)E_x(\omega_i)+E_y(\omega_s)E_y(\omega_i)\big)+d_{33}E_z(\omega_s)E_z(\omega_i)\Big].
\end{aligned}
\tag{1}
$$

where $E_{x,y,z}(\omega)$ denote the Cartesian components of either the signal electric field, at angular frequency ω_s, or the idler electric field, at angular frequency ($\omega_{\mathrm{i}}=\omega_{\mathrm{p}}-\omega_{\mathrm{s}}$), while ω_p is the pump angular frequency. The nonlinear coefficients are $d_{22}=1.9$ pm/V, $d_{31}=-3.2$ pm/V, and $d_{33}=\frac{1}{2}\chi^{(2)}_{zzz}=-19.5$ pm/V at 1313 nm, where the contracted notation, d, for $\chi^{(2)}$ was used.[2] As a first approximation the LN was considered with two sets of refractive indices, one at the wavelength of the resonance (≈ 1570 nm : $(n_{\mathrm{x}},n_{\mathrm{y}},n_{\mathrm{z}})=(2.21,2.21,2.14)$ and the other at the pump wavelength (788 nm) : $(n_{\mathrm{x}},n_{\mathrm{y}},n_{\mathrm{z}})=(2.26,2.26,2.18)$. The refractive index of the glass substrate is set to $n_g=1.44$, and the surrounding background is considered to be air.

1.2 Theoretical model

Assuming ideal detection, the photon-pair detection rate can be written as:[3,4]

$$\frac{\mathrm{d}^4 N_{\mathrm{pair}}}{\mathrm{d}t\,\mathrm{d}\Omega_{\mathrm{i}}\,\mathrm{d}\Omega_{\mathrm{s}}\,\mathrm{d}\omega_{\mathrm{s}}} = \mathcal{C}\left(n_{\mathrm{i}},\,\omega_{\mathrm{p}}-\omega_{\mathrm{s}};\,n_{\mathrm{s}},\,\omega_{\mathrm{s}}\right)\left|\widetilde{T}_{\mathrm{is}}(\theta_{\mathrm{i}},\varphi_{\mathrm{i}},\,\omega_{\mathrm{p}}-\omega_{\mathrm{s}},\,\mathbf{d}_{\mathrm{i}};\,\theta_{\mathrm{s}},\varphi_{\mathrm{s}},\,\omega_{\mathrm{s}},\,\mathbf{d}_{\mathrm{s}})\right|^2, \tag{2}$$

where

$$\mathcal{C}\left(n_{\mathrm{i}},\,\omega_{\mathrm{p}}-\omega_{\mathrm{s}};\,n_{\mathrm{s}},\,\omega_{\mathrm{s}}\right) \equiv \frac{8}{\pi\mu_0^2}\,n_{\mathrm{i}}n_{\mathrm{s}}\,\frac{(\omega_{\mathrm{p}}-\omega_{\mathrm{s}})^3\omega_{\mathrm{s}}^3}{{c_0}^6} \tag{3}$$

and

$$\widetilde{T}_{\mathrm{is}} = \sum_{\mathrm{m,n}=1}^{\infty} \xi_{\mathrm{m,n}}(\omega_{\mathrm{s}})\,\tilde{E}_{\mathrm{m},\mathbf{d}_{\mathrm{i}}}(\theta_{\mathrm{i}},\varphi_{\mathrm{i}})\,\tilde{E}_{\mathrm{n},\mathbf{d}_{\mathrm{s}}}(\theta_{\mathrm{s}},\varphi_{\mathrm{s}}). \tag{4}$$

is the complex two-photon amplitude, namely the probability amplitude for the joint detection of the idler and signal photons in the far-field directions specified by the spherical angles $(\theta_{\mathrm{i}},\varphi_{\mathrm{i}})$ and $(\theta_{\mathrm{s}},\varphi_{\mathrm{s}})$, with detection polarizations $\mathbf{d}_{\mathrm{i}}$ and $\mathbf{d}_{\mathrm{s}}$, respectively. The refractive indices of the surrounding medium at the idler and signal frequencies are denoted by n_{i} and n_{s}, whereas μ_0 and c_0 are the vacuum permeability and the speed of light in vacuum.

The detection rate is given per unit far-field solid angle, $d\Omega_{\mathrm{i}}d\Omega_{\mathrm{s}}$, and per unit signal-photon angular frequency, $d\omega_{\mathrm{s}}$. The quantity $\tilde{E}_{m,\mathbf{d}_{\mathrm{i}}}(\theta_{\mathrm{i}},\varphi_{\mathrm{i}})$ denotes the angular dependence of the complex far-field electric field of the m-th QNM, projected onto the detection polarization $\mathbf{d}_{\mathrm{i}}$. The summation runs over all QNM pairs (m,n), with the contribution of each pair weighted by the so-called modal-overlap coefficient

$$\xi_{\mathrm{m,n}}(\omega_{\mathrm{s}}) \equiv \frac{\mathcal{G}_{m,n}}{\mathcal{S}_{m,n}(\omega_{\mathrm{s}})}, \tag{5}$$

where

$$\mathcal{G}_{\mathrm{m,n}} = \sum_{\alpha,\beta,\gamma}\int d\mathbf{r}_0\,\chi^{(2)}_{\alpha\beta\gamma}(\mathbf{r}_0)\,\tilde{E}_{\mathrm{m},\alpha}(\mathbf{r}_0)\,\tilde{E}_{\mathrm{n},\beta}(\mathbf{r}_0)\,E_{\mathrm{p},\gamma}(\mathbf{r}_0) \tag{6}$$

and

$$\mathcal{S}_{\mathrm{m,n}}(\omega_{\mathrm{s}}) = (\omega_{\mathrm{p}}-\omega_{\mathrm{s}}-\tilde{\omega}_{\mathrm{m}})\tilde{\omega}_{\mathrm{m}}(\omega_{\mathrm{s}}-\tilde{\omega}_{\mathrm{n}})\tilde{\omega}_{\mathrm{n}}. \tag{7}$$

The integral $\mathcal{G}_{\mathrm{m,n}}$ quantifies the spatial overlap between the nonlinear susceptibility tensor $\chi^{(2)}$, the near-field QNM eigenfields $\tilde{\mathbf{E}}_{\mathrm{m}}$ and $\tilde{\mathbf{E}}_{\mathrm{n}}$ along the directions α and β, and the pump field $\mathbf{E}_{\mathrm{p}}$ along the direction γ. The spectral factor $\mathcal{S}_{\mathrm{m,n}}(\omega_{\mathrm{s}})$ accounts for the complex detuning between the generated photon frequencies $(\omega_{\mathrm{i}},\omega_{\mathrm{s}})$ and the QNM eigenfrequencies $(\tilde{\omega}_{\mathrm{m}},\tilde{\omega}_{\mathrm{n}})$, thereby determining the spectral and modal selectivity of SPDC.

The detected photon-pair count rate is calculated by integrating over all the range of angles allowed by the numerical aperture (NA) of the system and all the frequency

$$\frac{\mathrm{d}N_{\mathrm{pair}}}{\mathrm{d}t} = \int \mathrm{d}\omega_{\mathrm{s}}\int_{\mathrm{NA}}\mathrm{d}\Omega_{\mathrm{i}}\int_{\mathrm{NA}}\mathrm{d}\Omega_{\mathrm{s}}\,\mathcal{C}\left|\widetilde{T}_{\mathrm{is}}(\theta_{\mathrm{i}},\,\varphi_{\mathrm{i}},\,\omega_{\mathrm{p}}-\omega_{\mathrm{s}},\,\mathbf{d}_{\mathrm{i}};\,\theta_{\mathrm{s}},\,\varphi_{\mathrm{s}},\,\omega_{\mathrm{s}},\,\mathbf{d}_{\mathrm{s}})\right|^2, \tag{8}$$

where the variables entering the prefactor $\mathcal{C}$ have been omitted for brevity.

The QNMs are normalized according to the standard PML-based QNM normalization.[5,6] In particular, the electric and magnetic fields of each QNM are rescaled such that

$$\langle\tilde{\mathbf{E}}_m|\tilde{\mathbf{E}}_m\rangle = \int d\mathbf{r}\left[\tilde{\mathbf{E}}_m(\mathbf{r})\cdot\frac{\partial(\varepsilon(\omega)\,\omega)}{\partial\omega}\,\tilde{\mathbf{E}}_m(\mathbf{r}) - \tilde{\mathbf{H}}_m(\mathbf{r})\cdot\frac{\partial(\mu(\omega)\,\omega)}{\partial\omega}\,\tilde{\mathbf{H}}_m(\mathbf{r})\right] = 1, \tag{9}$$

where $\varepsilon(\omega)$ and $\mu(\omega)$ represent the permittivity and permeability of the domain, respectively, and the derivatives are evaluated at the complex eigenfrequency $\tilde{\omega}_m$. $\tilde{\mathbf{H}}_m(\mathbf{r})$ denote the magnetic-field profiles of the m-th QNM and the integral is performed over the whole simulated volume including the perfectly matched layers (PML). The correctness of this normalization is proven in the following transmission calculation.
In the main text since only the normal emission was considered the far-field $\widetilde{E}_z(\theta=0)$ was calculated as

$$\widetilde{E}_z(\theta=0) = \frac{1}{p^2}\int_{-p/2}^{p/2}\mathrm{d}y\int_{-p/2}^{p/2}\mathrm{d}z\,\widetilde{E}_z(x_1,y,z), \tag{10}$$

where p is the size of the unit-cell of the infinite periodical metasurface and x_1 denotes the position of the near-to-far transformation plane in the homogeneous air or substrate region.

2 Transmittance with QNMs

Here, we reconstruct the linear transmission spectrum of the periodic metasurface using an increasing number of QNMs, following the QNM expansion formalism,[5] and compared it with the transmission obtained directly from the full-wave simulation to verify the correctness of QNMs retrieval and normalization. The results are shown in Fig. S2. The left panel shows the complex eigenfrequencies of the QNMs considered in the reconstruction. The subset of nine modes providing the largest contributions to the transmitted field in the spectral range of interest is highlighted in red. The right panel compares the directly simulated transmission (black dots) with QNM reconstructions obtained using $M = 9$ modes (blue curve) and $M = 200$ modes (orange curve). As the number of QNMs is increased, the reconstructed transmission progressively approaches the full-field result. In particular, the reconstruction with $M = 200$ modes reproduces the position and spectral shape of the main resonant features.

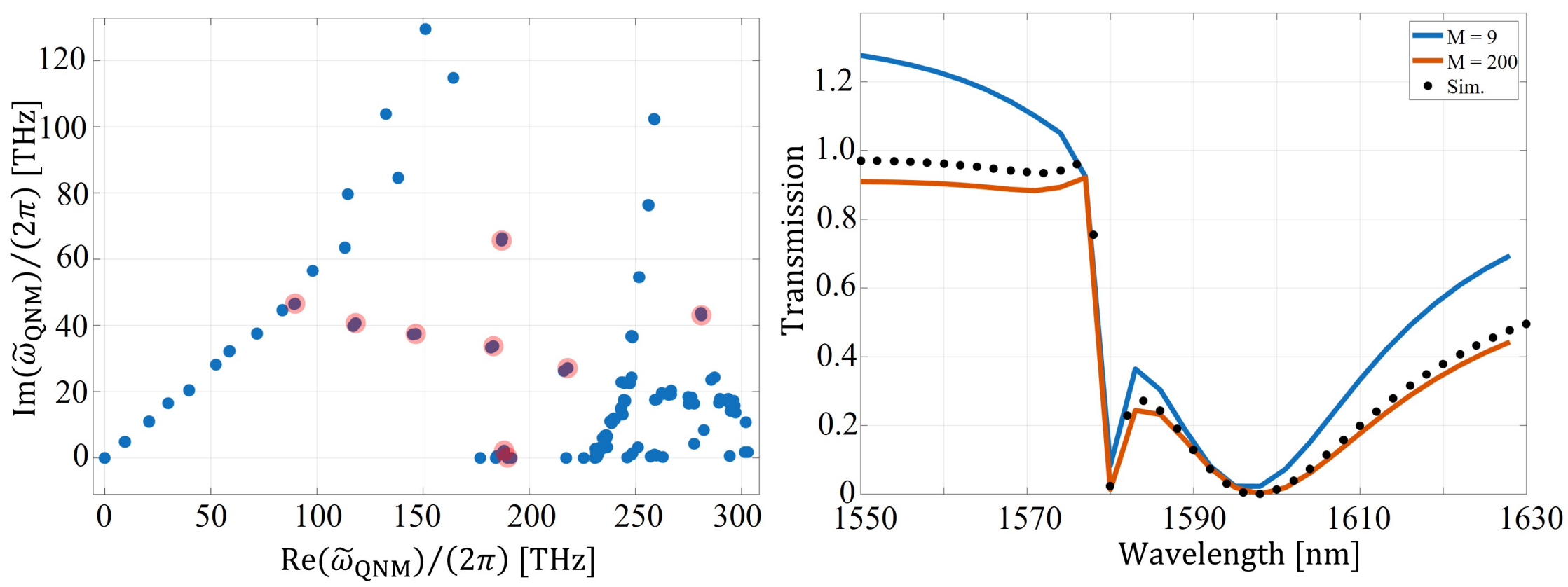

Figure S2: *Validation and convergence of the QNM reconstruction of the linear transmission.* Left: complex eigenfrequencies of the QNMs obtained from the eigenfrequency calculation. The nine modes providing the largest contributions to the transmitted field in the considered spectral region are highlighted in red. Right: comparison between the transmission obtained from the full-wave simulation (black dots) and from the QNM reconstruction using $M = 9$ (blue line) and $M = 200$ (orange line) modes.

As is generally encountered in QNM expansions of open electromagnetic systems, an accurate reconstruction of the linear response over a broad spectral range may require the inclusion of low-Q numerical modes associated with the PML discretization of the radiation continuum. These modes contribute primarily to the nonresonant background and improve the completeness of the linear-field expansion. These modes are the seven modes with imaginary part greater than 20. While the two modes with low imaginary part are the modes QNM_1 and QNM_2.

3 Modes with non-zero Floquet wave vector

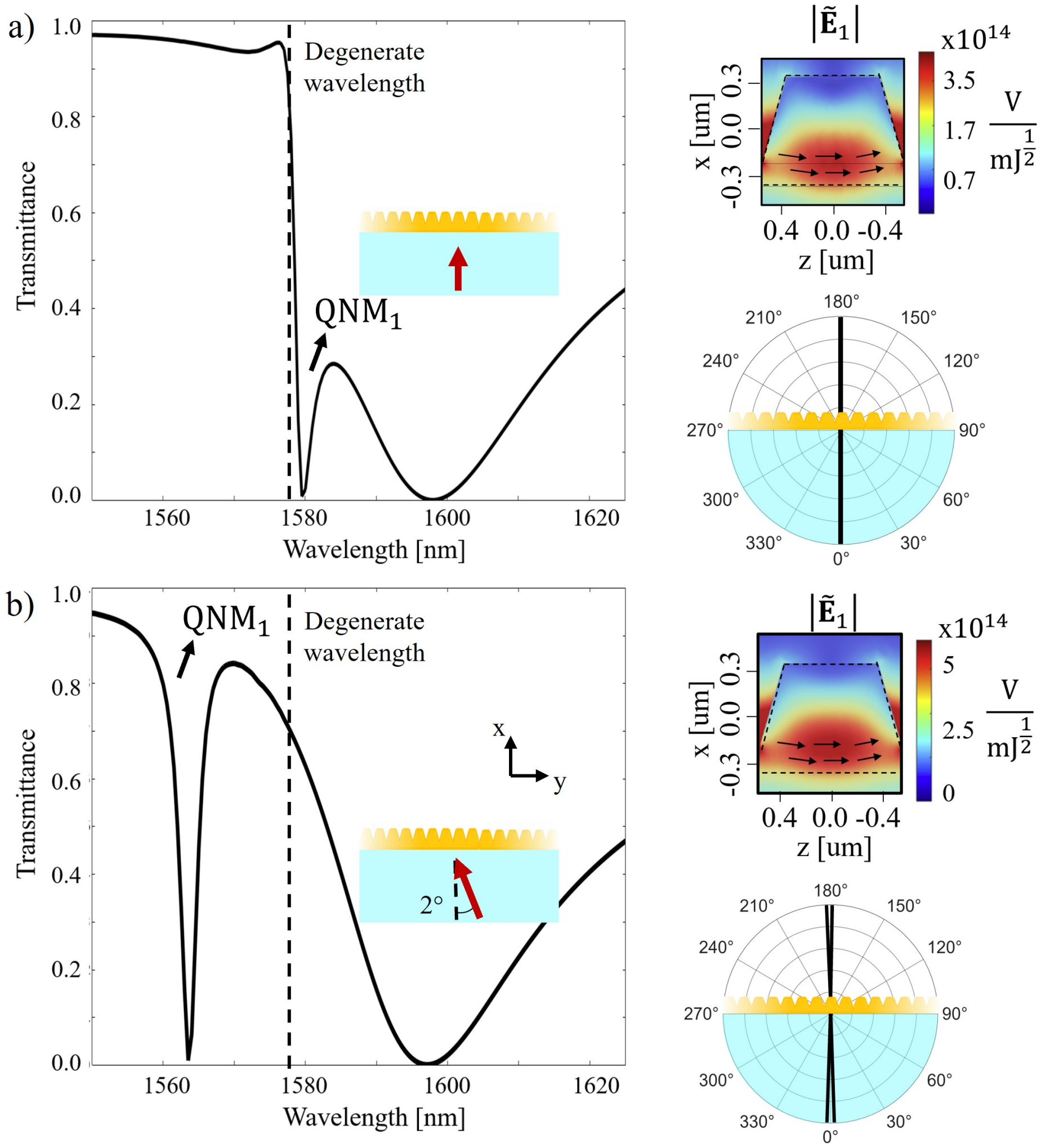

Figure S3: *Representative angle-dependent response of the QNM_1 resonance branch.* (a) Linear transmittance spectrum under normal incidence, corresponding to $\mathbf{k}_{\|} = 0$, together with the near-field and far-field radiation pattern of the associated Γ-point QNM. (b) Linear transmittance spectrum under oblique incidence at $\theta = 2°$, together with the QNM calculated using the corresponding nonzero Floquet wave vector. The near-field profile remains similar to that of QNM_1, indicating that the two modes belong to the same resonance branch, while the resonance wavelength and radiative channels are modified at nonzero $\mathbf{k}_{\|}$.

In the main text, the QNM analysis is restricted to the Γ-point resonance QNM_1, obtained with the COMSOL eigenfrequency solver by imposing a zero Floquet wave vector, $\mathbf{k}_{\|} = 0$. This corresponds to the normal-emission channel of the infinite periodic metasurface and provides the reduced mode-resolved description used in the main analysis to identify the physical mechanisms governing photon-pair generation and collection.

In a periodic metasurface, however, the QNM eigenfrequencies and radiative properties generally depend on the transverse wave vector. To illustrate this dependence, we performed angle-dependent linear-transmittance simulations and QNM calculations at nonzero Floquet wave vector. Fig. S3(a) shows the transmittance spectrum at normal incidence, where the narrow resonance dip is associated with QNM_1. Fig. S3(b) shows the corresponding response for oblique incidence at $\theta = 2°$. In this case, the resonance is shifted toward shorter wavelengths, consistently with the angle-dependent transmittance reported in the Supplementary Material of the original experimental work.[1]

The corresponding QNMs, calculated at $\mathbf{k}_{\parallel} = 0$ and at the nonzero Floquet wave vector associated with $\theta = 2°$, are shown on the right-hand side of Fig. S3. The off-Γ mode exhibits a near-field distribution similar to that of QNM_1, indicating that it belongs to the same resonance branch. At the same time, its complex eigenfrequency is shifted and its far-field radiation is redirected toward oblique channels.

This example shows that restricting the QNM description to $\mathbf{k}_{\parallel} = 0$ constitutes a reduced description of the periodic metasurface response. In a more general scenario, particularly when the strongest resonance is not at the degenerate wavelength with $\mathbf{k}_{\parallel} = 0$, QNMs at nonzero $\mathbf{k}_{\parallel}$ may become particularly relevant when their resonance wavelength approaches the degenerate SPDC wavelength, thereby minimizing the detuning term in Eq. (2) of the main text. A complete treatment of SPDC would therefore require the consistent inclusion of the relevant QNMs at nonzero $\mathbf{k}_{\parallel}$, together with their normalization, nonlinear modal overlaps, and radiative properties.

4 Finite size metasurface

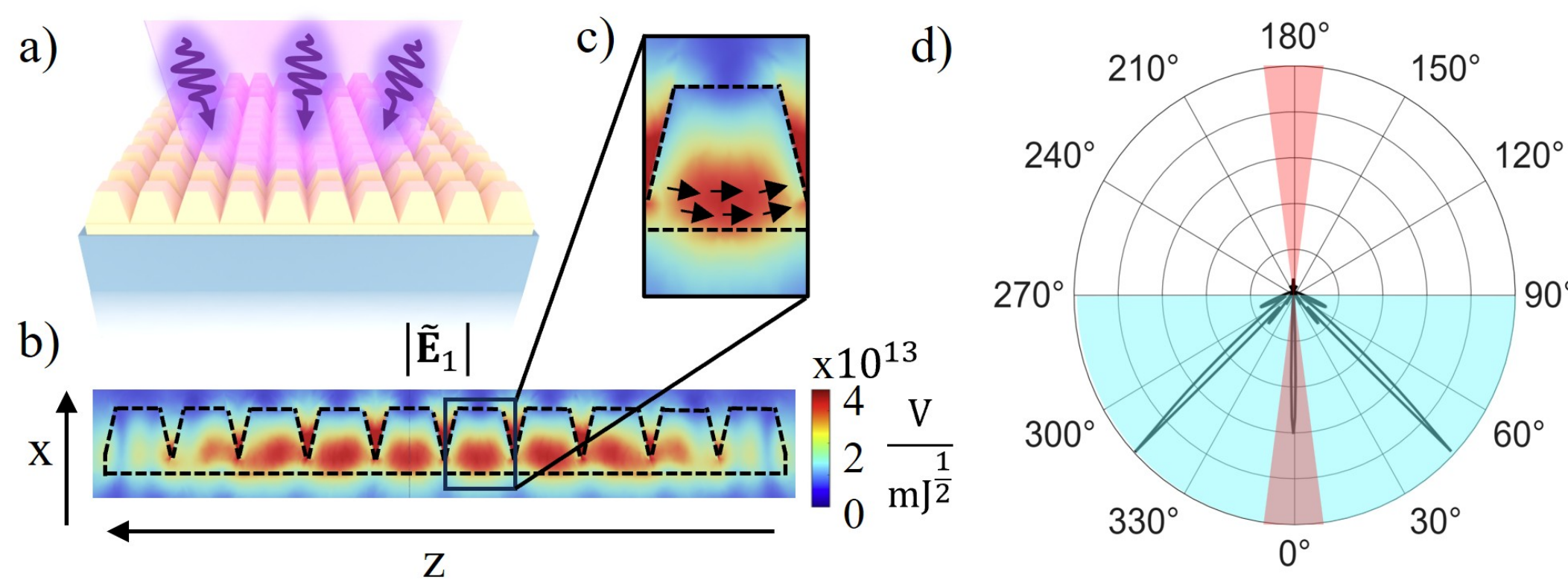

Figure S4: *Change of emission direction with a finite pump width* (a) The metasurface studied constis of only 10 × 10 unit-cells, simulating the effect of the experimental pump beam of 6 μm. The electric field of the resonance in the finite case is shown in (b) and in (c) a magnified view of the central unit-cell is shown. (d) The far-field of the corresponding resonance is shown, the blue area indicates the substrate while the red area indicates the angle that is acquired in the original experiment, determined by the numerical aperture of the fiber.

To assess the impact of the infinite-array approximation, we investigated the emission from a finite metasurface in which only a few unit cells are effectively excited, as schematically shown in Fig. S4 (a). This configuration mimics the experimental situation, where the finite pump waist illuminates only a limited number of resonators.

Fig. S4 (b) shows the near-field distribution of the corresponding finite-array mode, $|\tilde{\mathbf{E}}_1|$. The field retains the main features of the infinite-periodic QNM, with a strong electric field localized inside the LN resonators and predominantly polarized along the optical axis, i.e. the z-direction. This behavior is particularly clear in the central unit cells, shown in the zoom of Fig. S4 (c), which are less affected by the termination of the finite array.

The corresponding far-field emission pattern is shown in Fig. S4 (d), calculated according to.[7] In contrast to the infinite-periodic case, where the array factor strongly constrains the emission close to the metasurface normal, the finite array exhibits a trilobate angular spectrum with two lateral lobes in addition to the central one. This broadening arises from the relaxation of in-plane momentum selection and from radiation leakage associated with the finite edges of the structure. As a result, the mode can radiate over a wider range of angles.

However, most of the high-angle radiation lies outside the numerical aperture of the experimental collection system, indicated by the red shaded region in Fig. S4 (d). Therefore, only the emission close to the normal

direction contributes significantly to the detected signal. In this angular range, the finite array shows a pronounced asymmetry between the air and substrate half-spaces. This asymmetry originates from the combined effect of the finite excitation aperture and the refractive-index mismatch between the air and glass sides. Consequently, the emission collected from the air side is strongly suppressed compared to the substrate side, explaining why no measurable signal was observed experimentally in the air-side detection configuration.[1]

5 Substrate effects induced by diffraction channels

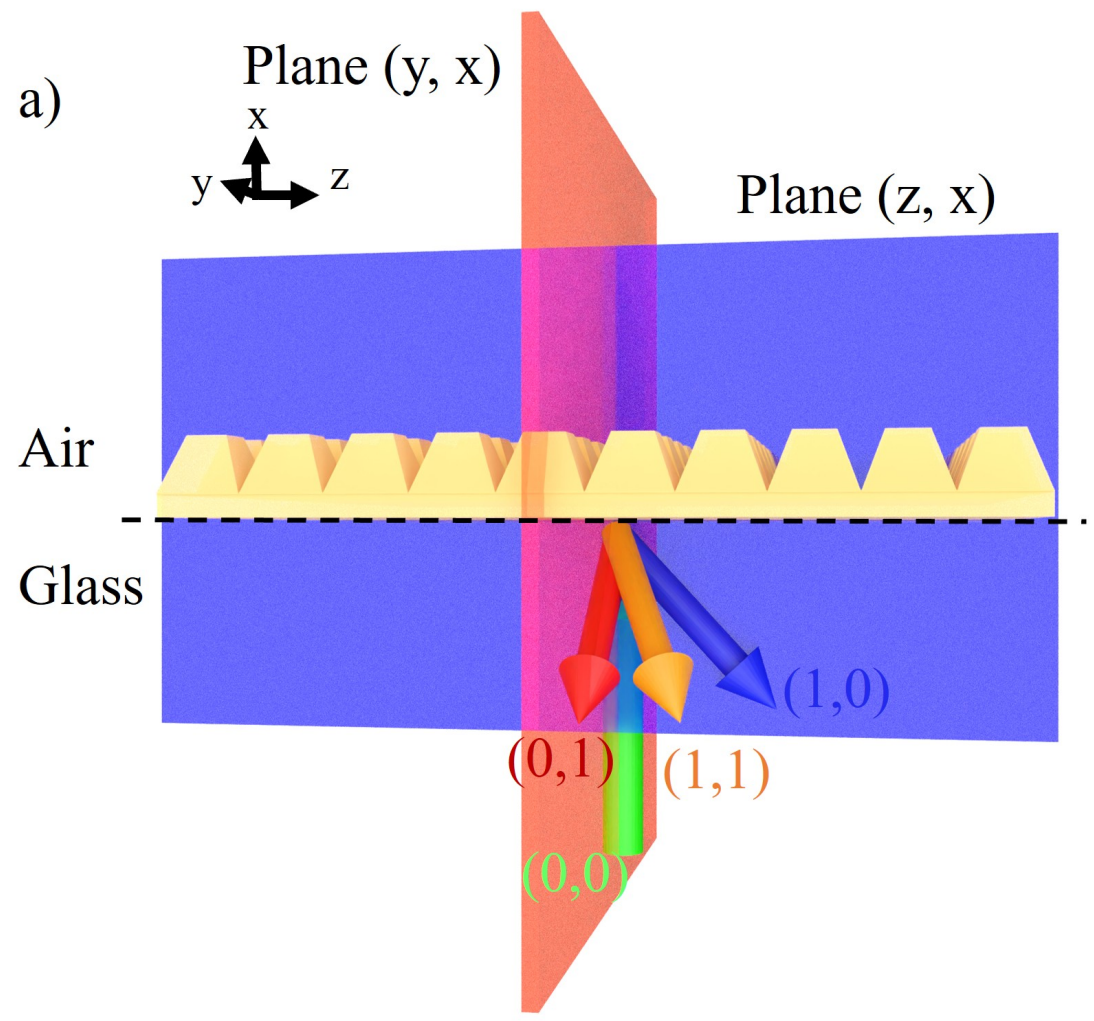

b) Total reflectance for incident channel (0,0): 0.44

(1,0) P	(-1,0) P	(0,1) S	(0,-1) S
0.09	0.09	0.02	0.02

(1,1) S	(-1,1) S	(1,-1) S	(-1,-1) S
0.05	0.05	0.05	0.05

Total reflectance for incident channel (-1,-1) S: 0.81

(-1,-1) S	(1,1) S	(0,0) S	(1,-1) S
0.20	0.18	0.13	0.05

(0,1) S	(0,-1) P	(-1,0) P	(0,1) P
0.03	0.04	0.06	0.03

Figure S5: *Pump diffraction orders generated by the metasurface.* (a) Schematic representation of the relevant pump diffraction orders inside the substrate. (b) Summary of the main reflectance coefficients associated with the most relevant diffraction channels.

The oscillatory behavior of the SPDC efficiency as a function of the substrate thickness can be understood as a finite-substrate interference effect involving the pump diffraction orders generated by the metasurface. Since the metasurface period is 900 nm, larger than the pump wavelength inside the glass substrate, the normally incident pump can be scattered into several propagating diffraction channels.

Fig. S5 (a) shows a schematic representation of the dominant diffraction orders generated inside the glass substrate. The zeroth-order channel $(0, 0)$ is shown in green, while three representative diffracted orders are highlighted in red $(0, 1)$, blue $(1, 0)$, and orange $(1, 1)$. For normal incidence, the total reflected power is approximately 0.44, while the remaining power is transmitted through the structure. The corresponding highest reflectance coefficients for the different diffraction orders and for polarizations parallel (P) and perpendicular (S) to the diffraction plane are reported in the upper table of Fig. S5 (b).

Owing to the symmetry of the metasurface under normal incidence, diffraction orders propagating in opposite transverse directions, such as $(-1, 0)$ and $(0, -1)$, exhibit the same reflectance coefficients. The dominant reflected contributions are associated with the $(\pm 1, 0)$ diffraction orders with P polarization, which propagate in the corresponding diffraction plane at an polar angle of approximately 37° with respect to the surface normal. Weaker contributions are associated with the $(0, \pm 1)$ orders with S polarization.

In addition, diffraction into the diagonal orders $(\pm 1, \pm 1)$ occurs at larger propagation angles, approximately 59°. Since this angle is larger than the critical angle at the glass–air interface, these orders undergo total internal reflection and are redirected back toward the metasurface. In contrast, for the diffraction orders propagating at $\theta \approx 37°$, the field is mainly transmitted through the glass-air interface for P polarization, while only about 0.2 of the incident power is reflected for S polarization.

To further investigate this mechanism, we calculated the diffraction response of the metasurface for an incident diagonal diffraction channel corresponding to the field of a diagonal order reflected back by the

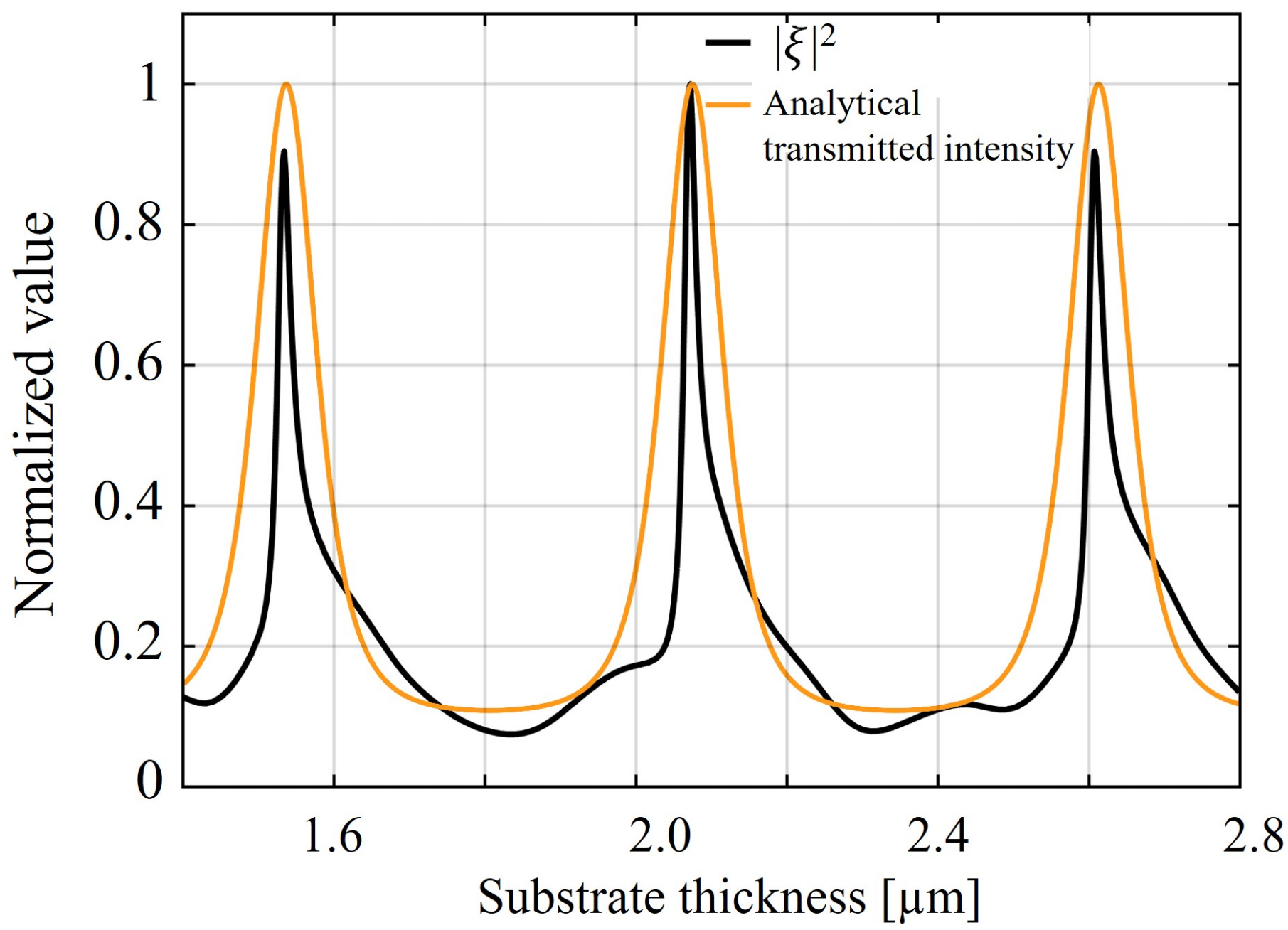

Figure S6: *Comparison between the simulated photon-pair generation efficiency and the analytical transmitted pump intensity.* The analytical transmitted intensity is obtained by considering the dominant pump diffraction orders and accounting for their propagation and multiple reflections inside the finite substrate. In particular, the model includes the main contribution from the diagonal diffraction orders $(\pm 1, \pm 1)$, which are responsible for the observed substrate-thickness-dependent modulation.

glass–air interface. The resulting highest diffraction efficiencies are reported in the lower table of Fig. S5 (b), where the orders are expressed in the global reference system. In this case, most of the incident power is reflected, with a total reflectance of approximately 0.81. The dominant reflected channels correspond to the same diagonal order and to the symmetry-related/specular diagonal channel, while a non-negligible contribution is also scattered into the zeroth order, that, however, is mainly transmitted in the glass–air interface.

These results show that the pump field dynamics inside the finite substrate are dominated by multiple reflections of the diffracted diagonal pump orders. Depending on the substrate thickness, the different reflected contributions acquire different propagation phases and interfere constructively or destructively at the metasurface position. This modifies the pump field coupled into the LN resonators and gives rise to the oscillatory behavior of the nonlinear overlap $|\xi|^2$, as shown in Fig. S6.

Based on these dominant diffraction channels, we constructed a simplified analytical model in which we retrieve the transmitted intensity of the pump as a function of the substrate thickness. The resulting transmitted pump intensity inside the nonlinear material is shown in Fig. S6 in the orange curve while the black curve is the $|\xi|^2$ shown in the main text. The model reproduces the main oscillation period observed in the full numerical simulations, confirming that the substrate-thickness dependence originates from the interference of diffracted pump orders.

The oscillation period is governed by the phase accumulated by a diffracted order during a round trip inside the substrate. For a diffraction order propagating at an angle θ inside the glass, this phase factor is

$$e^{2\, i\, k_0\, n_{\text{glass}}\, d\, \cos\theta}, \tag{11}$$

where $k_0 = 2\pi/\lambda_\text{p}$, n_{glass} is the refractive index of the substrate, and d is the substrate thickness. Constructive interference is therefore obtained when

$$2\, k_0\, n_{\text{glass}}\, d\, \cos\theta = 2\, \pi\, m, \tag{12}$$

with m an integer. The corresponding thickness period is

$$T = \frac{\lambda_{\mathrm{p}}}{2\, n_{\mathrm{glass}}\, \cos\theta}. \tag{13}$$

For the dominant diffraction channel considered here, this gives

$$T \simeq 573 \text{ nm}, \tag{14}$$

in agreement with the periodicity observed in the numerical simulations. The small shift of the peak positions and the residual modulations arise from additional diffraction channels, phase shifts upon reflection, and higher-order multiple-reflection processes that are not fully included in the simplified model.

For a more complete characterization, and to enable future quantitative comparisons, a full transfer-matrix-method approach could be implemented, following related studies of SHG in multilayer configurations.[8,9]

6 Proof-of-principle multilayer substrate design

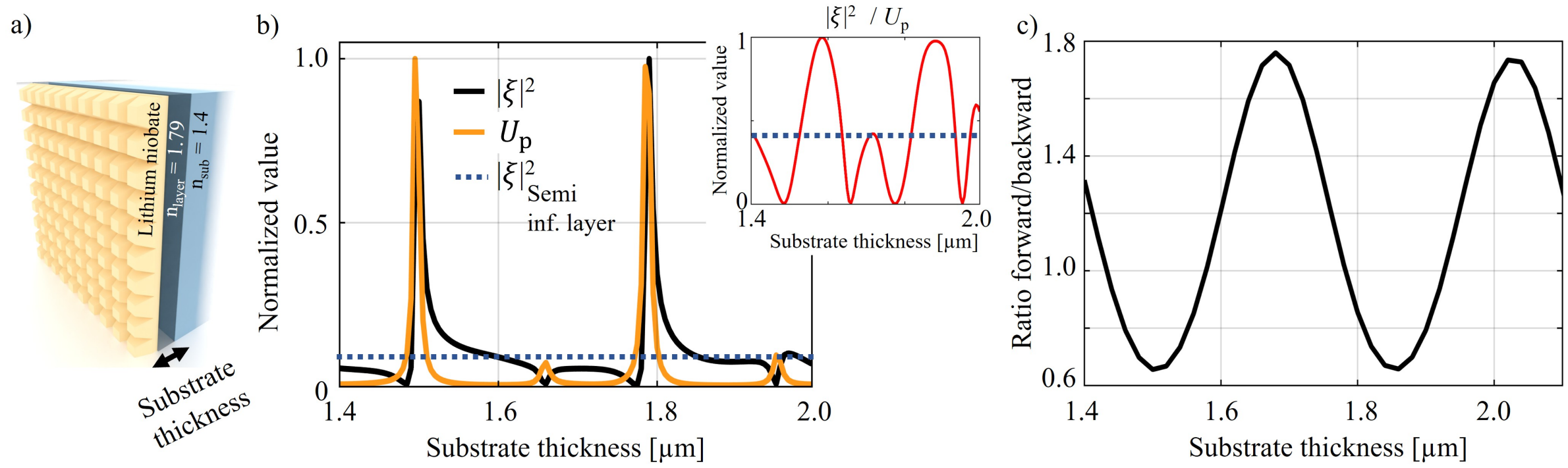

Figure S7: *Preliminary multilayer substrate design.*(a) Schematic of the multilayer geometry, consisting of an LN metasurface on a finite dielectric layer with refractive index $n \simeq 1.79$, followed by a semi-infinite glass-like substrate with $n \simeq 1.4$. The finite layer thickness is varied. (b) Normalized nonlinear-overlap contribution $|\xi|^2$ and integrated pump-field intensity U_{p} as a function of the finite-layer thickness. The blue dashed line indicates the corresponding semi-infinite-substrate reference value. The inset shows the normalized ratio $|\xi|^2/U_{\mathrm{p}}$. (c) Ratio between the forward and backward direction of the photon pairs as a function of the substrate thickness.

In the main text, we considered a finite air-backed glass substrate as an idealized geometry to isolate the physical mechanism responsible for substrate-induced enhancement, namely the recoupling and interference of pump diffraction orders. Here, we examine a preliminary multilayer configuration aimed at demonstrating that the same mechanism can, in principle, be implemented in a more realistic substrate geometry. This example is not intended to represent a fully optimized device, but rather a proof-of-principle demonstration of multilayer substrate engineering for enhancing the nonlinear overlap.

For realistic implementations, an important constraint must be considered. The diffraction orders responsible for the recoupling mechanism propagate obliquely inside the layer below the metasurface. Therefore, the reflecting or recoupling interface must be sufficiently close to the metasurface so that the reflected diffraction orders still spatially overlap with the illuminated region. In macroscopic substrates, the lateral displacement of these oblique orders can become much larger than the pump-beam waist, strongly reducing their ability to recouple to the metasurface. This motivates the use of a finite optical layer with a thickness of the order of a few micrometers, followed by an additional substrate or multilayer structure.

The multilayer geometry considered here is shown in Fig. S7 (a). The LN metasurface is deposited on a finite dielectric layer with refractive index $n_{\mathrm{layer}} \simeq 1.79$, representative of a sapphire-like material, followed

by a semi-infinite glass-like substrate with refractive index $n_{\text{sub}} \simeq 1.4$. This configuration preserves a finite optical path below the metasurface while avoiding the idealized air-backed substrate considered in the main text.

We emphasize that, in this proof-of-principle example, the resonator geometry was redesigned with respect to the original telecom-wavelength metasurface. The purpose of this redesign is to place the relevant pump diffraction orders in a regime where they can be internally reflected at the interface between the finite dielectric layer and the lower-index substrate. For a diffraction order (m, n), the propagation angle inside the finite layer is determined by

$$\sin\theta_{\text{mn}} = \frac{\lambda_{\text{p}}}{n_{\text{layer}}\, p}\sqrt{m^2 + n^2}, \tag{15}$$

where λ_{p} is the pump wavelength and p is the metasurface period. Internal reflection at the interface with the lower-index substrate requires

$$\sin\theta_{\text{mn}} > \frac{n_{\text{sub}}}{n_{\text{layer}}}. \tag{16}$$

Therefore, efficient multilayer-enhanced recoupling is not automatic, but requires simultaneous engineering of the lattice period, pump wavelength, and refractive-index contrast.

To satisfy this condition, the resonator geometry was redesigned to support the dominant QNM around the degenerate wavelength $\lambda_{\text{s}} = \lambda_{\text{i}} \simeq 1250$ nm, corresponding to a pump wavelength $\lambda_{\text{p}} \simeq 625$ nm. The optimized truncated-pyramid geometry has period 615 nm, height 864 nm, a bottom-to-top base ratio of 0.64, and a residual layer thickness of 420 nm. This geometry is used as a representative example rather than as a fully optimized structure. Similar multilayer-enhancement strategies could in principle be implemented at other wavelengths by redesigning the resonator geometry, choosing a different nonlinear material platform, or engineering a suitable substrate stack.

The results are shown in Fig. S7 (b). As in the finite-substrate analysis discussed in the main text, the nonlinear-overlap contribution $|\xi|^2$ exhibits pronounced maxima as a function of the finite-layer thickness. These maxima closely follow the peaks of the integrated pump-field intensity inside the nonlinear material, U_{p}, confirming that the enhancement is mainly associated with constructive recoupling of the pump field into the LN resonators. Compared to the reference semi-infinite substrate with refractive index $n = 1.79$, chosen by having the same resonance frequency, indicated by the blue dashed line, the multilayer configuration provides an enhancement of more than one order of magnitude in $|\xi|^2$.

Importantly, the enhancement is not solely due to a larger pump-field intensity inside the nonlinear material. As shown in the inset of Fig. S7 (b), the normalized ratio $|\xi|^2/U_{\text{p}}$ also exhibits clear thickness-dependent maxima. A maximum improvement of approximately a factor of two is obtained compared to the corresponding semi-infinite reference case. This indicates that the multilayer substrate can improve the coherent nonlinear overlap, and not only the amount of pump field coupled into the LN resonators.

Furthermore, Fig. S7 (c) shows that the multilayer geometry also induces oscillations in the forward/backward emission ratio. These oscillations arise from etalon-like interference effects inside the finite dielectric layer, which modify the relative photon-pair emission into the two directions.

References

(1) Santiago-Cruz, T.; Fedotova, A.; Sultanov, V.; Weissflog, M. A.; Arslan, D.; Younesi, M.; Pertsch, T.; Staude, I.; Setzpfandt, F.; Chekhova, M. Photon pairs from resonant metasurfaces. *Nano letters* **2021**, *21*, 4423–4429.

(2) Shoji, I.; Kondo, T.; Kitamoto, A.; Shirane, M.; Ito, R. Absolute scale of second-order nonlinear-optical coefficients. *JOSA B* **1997**, *14*, 2268–2294.

(3) Poddubny, A. N.; Iorsh, I. V.; Sukhorukov, A. A. Generation of photon-plasmon quantum states in nonlinear hyperbolic metamaterials. *Physical review letters* **2016**, *117*, 123901.

(4) Weissflog, M. A.; Dezert, R.; Vinel, V.; Gigli, C.; Leo, G.; Pertsch, T.; Setzpfandt, F.; Borne, A.; Saravi, S. Nonlinear nanoresonators for Bell state generation. *Applied Physics Reviews* **2024**, *11*, DOI: `https://doi.org/10.1063/5.0172240`.

(5) Lalanne, P.; Yan, W.; Vynck, K.; Sauvan, C.; Hugonin, J.-P. Light interaction with photonic and plasmonic resonances. *Laser & Photonics Reviews* **2018**, *12*, 1700113.

(6) Sauvan, C.; Wu, T.; Zarouf, R.; Muljarov, E. A.; Lalanne, P. Normalization, orthogonality, and completeness of quasinormal modes of open systems: the case of electromagnetism. *Optics Express* **2022**, *30*, 6846–6885.

(7) Yang, J.; Hugonin, J.-P.; Lalanne, P. Near-to-far field transformations for radiative and guided waves. *ACS photonics* **2016**, *3*, 395–402.

(8) Pakhomov, A. V.; Hammerschmidt, M.; Burger, S.; Pertsch, T.; Setzpfandt, F. Modeling of surface-induced second-harmonic generation from multilayer structures by the transfer matrix method. *Optics Express* **2021**, *29*, 9098–9122.

(9) Wang, W.; Song, Y.; Shan, Y.; Sipe, J.; Cheng, J. L. Theory of the interference tunability of second harmonic generation for two-dimensional materials in layered structures. *Optics Express* **2024**, *32*, 31495–31513.
